# Optimizing autofocus under multispectral lighting via enhanced SIFT and Pearson correlation coefficient

**DOI:** 10.1371/journal.pone.0336810

**Published:** 2025-11-17

**Authors:** Chao Ma, Mingkun Zhang, Zhiyong Dai, Qiuyu Zhang, Jianwei Ma, Xiaolin Niu, Yongyi Yan

**Affiliations:** 1 College of Information Engineering, Henan University of Science and Technology, Luoyang, China; 2 Complex System Intelligent Control Laboratory, Shanghai Jiao Tong University, Shanghai, China; 3 College of Cyber Science and Engineering, Xi’an Jiaotong University, Xi’an, China; University of the Punjab, PAKISTAN

## Abstract

The use of inexpensive black and white cameras in conjunction with multi-band lighting offers the highest accuracy and cost benefits among the several techniques for accomplishing multispectral images. The focus point shifts with varied wavelength illumination, since the lens optical glass has varying refractive indices for different wavelength light sources. Thus, quick and precise focusing is essential for enhancing system efficiency as a whole. To solve this problem, this study proposes a multispectral quick focusing method. First, analyzes the current methods for evaluating image sharpness and proposes an improved Tenengrad function for the focused scene that could extract gradient information from the image in several directions and improve sharpness evaluation. The improved gradient extraction method combines the Scale Invariant Feature Transformation (SIFT) algorithm to form a new multi-scale image sharpness evaluation function, SIFT Quad-Tenen. To improve the focusing efficiency and optimize the focusing process, a search strategy combining a climbing search algorithm and a traversal method was proposed. Finally, considering the similarity of images between different wavelength bands under multi-light source conditions, the Pearson correlation coefficient is introduced to improve the focusing speed and accuracy. The experimental results demonstrate the superiority of the SIFT Quad-Tenen evaluation function in terms of stability and sensitivity, as well as the significant improvement of the focusing speed and accuracy of the Pearson-Hill climbing algorithm.

## 1 Introduction

Spectrum is the collection of monochromatic light arranged in an orderly manner according to the wavelength after being decomposed by a dispersive system (such as a prism or grating). It includes the invisible infrared and ultraviolet rays in addition to the visible light spectrum, which is discernible to the human eye. The measurement of a substance’s spectral reflectance, or its reflection to a particular band of light, provides information about its physicochemical characteristics and has grown in importance as an optical detection method [1–4]. With the advent of multispectral imaging technology in the field of satellite remote sensing since the late sixties of the last century, this technology has steadily extended to agricultural monitoring, food safety, medical imaging, and safety monitoring from the initial remote sensing [5,6]. It has excellent analytical capabilities and is also widely used in computer vision and image processing [7,8]. Multispectral imaging technology has promoted the development of multispectral imaging equipment, which can more effectively collect and analyze spectral data and offer robust support for multispectral technology’s multi-field application [9–12].

Combining the capabilities of target imaging with spectrum acquisition, the multispectral imaging device is a commonly used detection tool [1,13]. It can combine the target’s spectral and spatial information, conduct spectral imaging of the same object in the continuous spectral band, and greatly enhance the information dimension of target observation [8,14]. Depending on how devices work, these devices fall into four major categories: computational multispectral cameras, dispersive, filter, and interferometric cameras [15–17]. To achieve spectrum separation and imaging, dispersive multispectral cameras use a prism or grating as a dispersive component [18]; Filter type multispectral cameras are equipped with a rotatable narrow-band filter wheel in front of a normal camera to selectively capture images in a specific wavelength band [19]; Interferometric multispectral cameras use the interferometric principle to obtain spectral information of targets by measuring the interference patterns at different optical path differences and performing the reverse Fourier transform [20]. Computational multispectral cameras, on the other hand, use advanced algorithms to reconstruct spectral images from the acquired data. As a result of these technological advancements, multispectral imaging devices have become commonplace in both industrial and research [15,21,22]. However, every type of multispectral imaging device has specific characteristics and limitations.

Dispersive multispectral cameras offer high spectral resolution and continuous spectral acquisition, making them suitable for applications requiring fine spectral analysis in the field of multispectral imaging technology [7,23]. However, their systems are large, complex, expensive, and slow to image; sometimes the Signal-to-Noise Ratio (SNR) is decreased due to uneven light intensity distribution [18,24,25]. In contrast, filter-based multispectral cameras are widely favored due to their simple structure, low cost, fast imaging speed, and easy miniaturization [26,27], but their spectral resolution is limited by the choice of filters, and the filter configuration is difficult to change once fixed [28,29]. Interferometric multispectral cameras can provide extremely high spectral resolution, but they are highly complex, require strict maintenance and calibration, and require complex algorithms to reconstruct spectral images [29,30]. Computational multispectral cameras can flexibly adjust spectral channels through software algorithms, and the hardware design tends to be miniaturized and integrated, and the cost-effectiveness increases with the increase of computing power [31]. Therefore, the multispectral imaging system based on Light Emitting Diodes (LED) strobe computational imaging has become an attractive solution.

LEDs are used as the light source in the multispectral imaging system based on LED strobe computational imaging. The target is illuminated by progressively regulating the emission of light from LEDs of various wavelengths. The capability of this camera to accurately regulate the sequence and timing of the LEDs’ opening at each wavelength allows it to image the target sequentially under various light wavelengths. After the images of each wavelength are captured by the camera’s sensor, these images are reconstructed into a multispectral image by a processing algorithm. The main advantages of this approach are its flexible light source control, low cost, and low energy consumption, making it suitable for portable or miniaturized multispectral imaging systems. However, since different wavelengths of light sources can cause changes in the refractive index of the imaging system, affecting the positive focus position of the imaging [32], the imaging system needs to have the ability to autofocus under different wavelength light sources.

An enhanced Tenengrad evaluation function is presented to increase the accuracy of image sharpness evaluation by incorporating multi-directional gradient information extraction, which improves the focusing speed and accuracy of cameras under various wavelength light sources. At the same time, the SIFT algorithm is introduced to improve the anti-interference ability of multi-light source scenes. In terms of search strategy, the improved hill-climbing search algorithm was selected, the focus area was determined by coarse focusing, and then the ergodic method was used to fine-tune the focus in the focus area, and the Pearson correlation coefficient was added to determine the lighting sequence, to further optimize the search strategy. Finally, a multispectral illumination autofocus experimental platform is constructed that can simulate scenes of various band light sources to evaluate and improve the autofocus algorithm’s performance under various band light sources.

## 2 Related work

### 2.1 Image sharpness evaluation

In the process of realizing autofocus, the autofocus algorithm needs to rely on the image sharpness evaluation function to determine whether to focus or not. At present, image sharpness evaluation methods are mainly divided into three categories: spatial domain, frequency domain, and deep learning [15]. Frequency domain methods evaluate the sharpness of an image by analyzing its frequency components, and commonly used techniques include the Fourier transform [18] and wavelet transform [19]. However, these methods are rarely used in practical applications due to their high computational complexity and poor real-time performance. Deep learning-based methods for evaluating image sharpness have steadily gained popularity as deep learning has advanced. The deep learning methods train a network model on a dataset and input the image to be detected into the network to obtain an image sharpness value. This method relies on a large number of datasets for training, and it performs well in image feature extraction and model training [20], but its operation is complex, and the real-time performance is poor. The spatial domain evaluation approach analyzes the relationship between image pixels and adjacent pixels to distinguish between clear and blurry images. Common spatial domain evaluation functions include the Roberts function, Brenner function, and Tenengrad function. These methods are simple to calculate and efficient, so they are widely used in practical applications. However, the traditional spatial domain method is sensitive to noise and has poor noise immunity. To improve the noise immunity of these methods, the researchers made several improvements. Zhan et al. evaluated the quality of blurred images by combining maximum gradient and gradient variability with subjective scoring [33]. Li et al. obtained image sharpness values by dividing the gradient image of the image into blocks of equal size and using an orthogonal matrix [34]. Zhang et al. used the Sobel operator to obtain the gradient information in the four directions of the image, and combined it with the Brenner function, proposed an improved autofocus algorithm with the Sobel function [35]. Xiong et al. improved the Brenner function to obtain gradient information in more directions and improve the accuracy of image sharpness evaluation [36].

### 2.2 Search strategy

In the autofocus algorithm, search strategies are needed to find the positive focal length, and the main search strategies include traversal search, Fibonacci search, curve fitting search, and hill climbing search. The traversal search method determines the optimal focal length by evaluating the sharpness of the image over the entire focal length range. The Fibonacci search method uses the Fibonacci sequence to efficiently narrow the search area [37]. The curve fitting search method predicts the optimal focal length point by fitting the curve of the relationship between image sharpness and focal length. The traversal search method is simple and can ensure the global optimal solution, but it is inefficient. The Fibonacci search method is efficient and predictable, but it may fall into local optima. The curve fitting search method improves the speed and accuracy through model prediction, but requires sufficient data and a suitable model. The hill climbing search method starts from the initial point and gradually moves to the direction of increasing the value of the evaluation function in the neighborhood, and The hill-climbing search method responds quickly and is simple to implement, but it is easily affected by the local optimal solution and the initial point selection. Guo et al. [38] proposed a sharpness evaluation function based on histogram eigenfunction and a hill climbing search algorithm judged by slope change. Wu et al. [39] proposed a partition search algorithm combining adaptive step size and a two-step search method to improve the anti-interference ability of the algorithm. Jia et al. [40] divided the improved hill climbing search algorithm into coarse focusing methods based on small focus areas and fine focusing methods based on function approximation to improve focusing accuracy.

## 3 Proposed optimization algorithm

### 3.1 SIFT Quad-Tenen evaluation function

**Fig 1:**
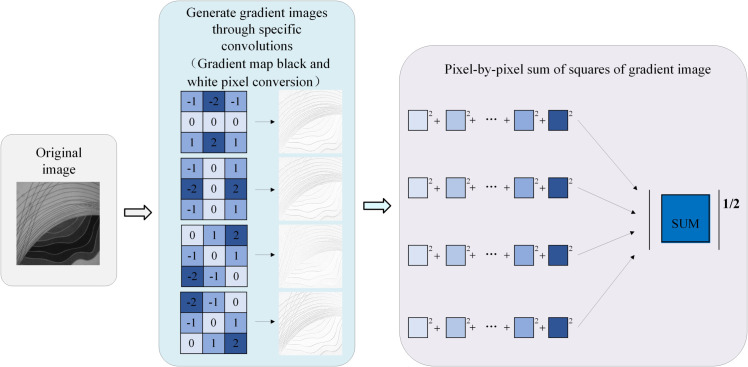
Multi-directional image sharpness evaluation algorithm.

The traditional Tenengrad function uses the Sobel operator to extract the horizontal and vertical gradients of an image. Although the traditional Tenengrad function is widely used in image sharpness evaluation, it mainly extracts the horizontal and vertical gradient information of the image and ignores the gradient in other directions. This limitation may result in the Tenengrad function not providing an accurate assessment of sharpness when evaluating images with complex textures or oblique features [16]. To solve this problem, as shown in Fig 1, the algorithm proposed in this study improves the Tenengrad function and adds gradient information extraction ± 45^°^ direction. This improvement allows the algorithm to obtain gradient information from multiple directions, to more accurately evaluate the sharpness value of the image. The improved function uses the Sobel operator template in four directions, corresponding to 0^°^, 45^°^, 90^°^, and 135^°^ directions, respectively, to achieve comprehensive capture of image gradient information. The improved function uses the Sobel operator template in four directions, which are expressed as Eq 1:

$$\begin{array}{cc} S_{1}=\left[\begin{array}{ccc} -1 & -2 & -1\\ 0 & 0 & 0\\ 1 & 2 & 1 \end{array}\right] & S_{2}=\left[\begin{array}{ccc} -1 & 0 & 1\\ -2 & 0 & 2\\ -1 & 0 & 1 \end{array}\right]\\ S_{3}=\left[\begin{array}{ccc} 0 & 1 & 2\\ -1 & 0 & 1\\ -2 & -1 & 0 \end{array}\right] & S_{4}=\left[\begin{array}{ccc} -2 & -1 & 0\\ -1 & 0 & 1\\ 0 & 1 & 2 \end{array}\right] \end{array}$$ (1)

*S*_1_ and *S*_2_ are the Sobel operator templates in the horizontal and vertical directions, and *S*_3_ and *S*_4_ are the Sobel operator templates in the ±45^°^ direction. Although the improved Tenengrad adopts the strategy of extracting gradient information in 4 directions, the improved function considers 8 adjacent pixels of (x,y) pixels. The improved expression for extracting the multidirectional gradient information for the Tenengrad function (Quad-Tenengrad function) is Eq 2:

$$F_{Q-T}=\sqrt{I_{1}{\left(x,y\right)}^{2}+I_{2}{\left(x,y\right)}^{2}+I_{3}{\left(x,y\right)}^{2}+I_{4}{\left(x,y\right)}^{2}}$$ (2)

where *I*_1_(*x*,*y*), *I*_2_(*x*,*y*), *I*_3_(*x*,*y*), and *I*_4_(*x*,*y*) are Eq 3:

$$\{\begin{array}{c} I_{1}(x,y)=f(x,y)*S_{1}\\ I_{2}(x,y)=f(x,y)*S_{2}\\ I_{\text{3}}(x,y)=f(x,y)*S_{\text{3}}\\ I_{\text{4}}(x,y)=f(x,y)*S_{\text{4}} \end{array}$$ (3)

When extracting gradient information in four directions, the Quad-Tenengrad function may introduce additional noise information. To suppress these noises, a weight allocation strategy based on image texture structure information was proposed. When calculating the image sharpness value, the algorithm will adjust the weight according to the richness of texture structure information in different areas of the image. Areas of the image that are rich in texture structure information will be given higher weight, while areas lacking texture information will be given lower weight. This method can not only suppress the extraction of noise information but also enhance the algorithm’s ability to recognize effective texture information of the image, to improve the anti-noise performance of the algorithm.

Scale-invariant feature transform (SIFT) is a feature extraction technology widely used in the field of image processing, which can extract feature points that are invariant to rotation, scale scaling and brightness changes, and has good robustness to noise. The SIFT algorithm is applied to the local area of the image to improve the performance of the Quad-Tenengrad function, and the weight of the feature points is assigned accordingly. The key steps of the SIFT algorithm are as follows:

(1) Scale-space construction: The Gaussian difference pyramid is used to generate a scale space, which involves applying Gaussian filters of different scales to the original image to obtain a series of images that are gradually blurred. The mathematical expression is Eq 4:

L(x,y,σ)=G(x,y,σ)*f(x,y) (4)

where L represents the scale space function, f is the input image, G is the Gaussian function, *σ* is the scale coordinate, and its function form is Eq 5:

$$G(x,y,\sigma )=\frac{1}{2\pi \sigma^{2}}\exp\left(-\frac{x^{2}+y^{2}}{2\sigma^{2}}\right)$$ (5)

Through the further processing of the Gaussian pyramid, the Difference of Gaussian is established, and the scale space is established. The function form is as Eq 6:

D(x,y,σ)=[G(x,y,kσ)−G(x,y,σ)]·f(x,y)=L(x,y,kσ)−L(x,y,σ) (6)

(2) Feature point detection: In the scale space, by comparing the target pixel with the pixel in the neighborhood, the extreme point is identified as the key point. According to the distribution characteristics of pixels in the vicinity of the key points in the gradient direction, the gradient method is used to ensure that the filtered feature points have rotation invariance when rotating. The pixel (x,y) direction and amplitude are represented by *m*(*x*,*y*), θ(x,y), Eqs 7 and 8.

$$m(x,y)=\sqrt{{\left(L(\left(\text{x}+1),\text{y}\right)-L(\left(\text{x}-1),\text{y}\right)\right)}^{2}+{\left(L(x,(y+1))-L(x,(y-1))\right)}^{2}}$$ (7)

$$\theta (x,y)=\arctan\frac{L(x,(y+1))-L(x,(y-1))}{L((x+1),y)-L((x-1),y)}$$ (8)

where *L*(*x*,*y*) is the scale space where pixel (*x*,*y*) is located.

(3) Generate descriptors: After determining the orientation of the key, the gradient information of the pixels around the key is used to generate the feature descriptor. The feature description mainly includes the following steps: determining the neighborhood region of the key point, dividing the neighborhood region into several subregions, calculating the gradient direction histogram for each subregion, connecting the gradient direction histograms of all subregions to form a feature vector, and the feature descriptor obtained by the SIFT algorithm is a 128-dimensional vector. Weights are assigned according to the number of feature points in each image, and the formula for calculating weight a is as Eq 9:

$$a_{i}=\frac{N_{i}}{N_{t\times t}}$$ (9)

where *N*_*i*_ is the number of feature points of the ith image, and $N_{t\times t}$ is the number of feature points of the whole image. Then the formula for the SIFT Quad-Tenen function is Eq 10.

$$F_{SIFTQuad\_Tenen}=\sqrt{I_{1}{\left(x,y\right)}^{2}+I_{2}{\left(x,y\right)}^{2}+\sum_{i=1}^{t\times t}a_{i}\left[I_{3}{\left(x,y\right)}^{2}+I_{4}{\left(x,y\right)}^{2}\right]}$$ (10)

**Fig 2:**
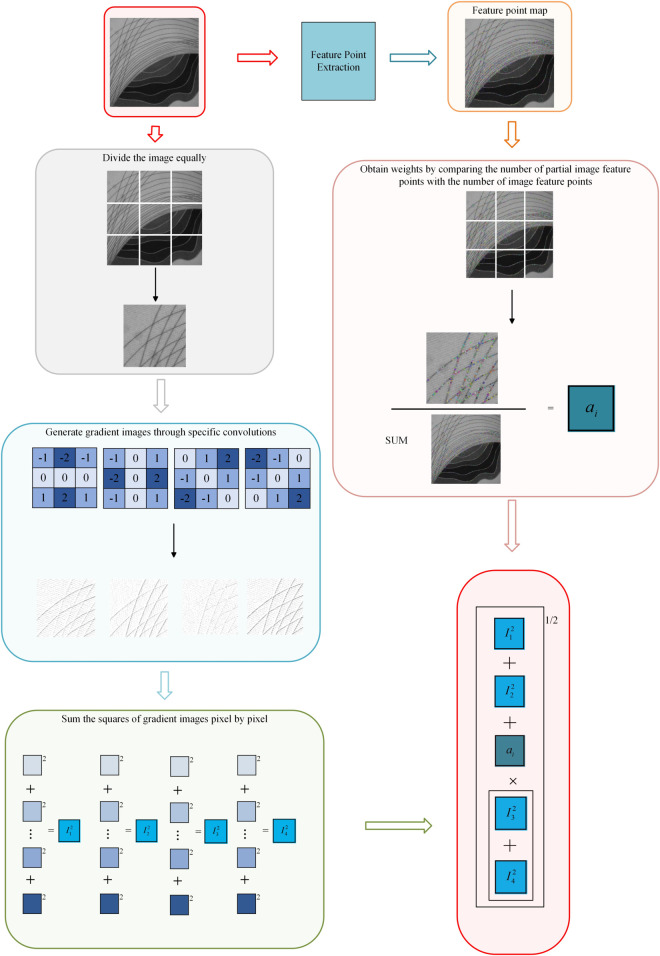
SIFT Quad-Tenen diagram.

As shown in Fig 2, SIFT Quad-Tenen function is an image sharpness evaluation method that combines SIFT feature point detection and Quad-Tenengrad multi-directional gradient information extraction. This function has significant anti-interference characteristics during autofocus, especially in scenes where different light sources are switched.

### 3.2 Pearson hill climbing algorithm

The hill climbing search algorithm is widely used in the search strategy of camera focus, but it has some limitations in the focus search process, especially in terms of speed. The hill climbing algorithm may need to be iterated several times to gradually approach the optimal solution, and the focusing speed does not meet the background requirements. Therefore, the hill climbing algorithm was changed to a coarse-fine hill climbing algorithm by combining the characteristics of the traversal method.

**Fig 3:**
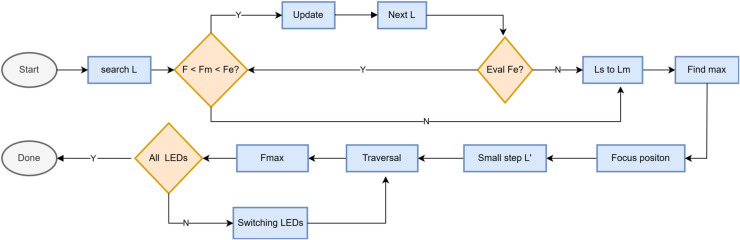
Coarse-fine optimization algorithm.

The process of the algorithm is as shown in Fig 3.

(1) Starting from the starting position, the search was started with the large step size L, and the images of the starting position, step length L and 2L were evaluated respectively, and the sharpness values were F, Fm, and Fe.

(2) Compared with F, Fm and Fe, when Fs <Fm<Fe proves that the search direction is correct, the new Fs is assigned Fm and the new Fm Fe, and the next step L image is searched to obtain the new evaluation value and assigned to Fe.

(3) Compare the new F, Fm, and Fe, and continue to step 2 if Fs < Fm < Fe. If Fs < Fm and Fe<Fm, step 4 is performed on the interval from the focal length of Fs to the area where Fm is located Ls, Lm.

(4) For the interval Ls, Lm, the traversal method is used to obtain the maximum image sharpness value, and the focal length where the maximum value is located is the positive focal position.

(5) The remaining light source scenes are centered on the focal length of the previous scene, and the focus is completed by traversing and searching Fmax in small steps L’ on the left and right sides of the focal length position.

The improved hill climbing algorithm improves the focusing speed, but the positive focus position of different light source scenes cannot be guaranteed to be within the step size L’, and the Pearson correlation coefficient is introduced to optimize the coarse-fine hill climbing algorithm to ensure the focusing accuracy. Pearson correlation coefficient let the two sets of data be X and Y, and calculate the mean values $\overset{―}{\text{X}}$ and $\overset{―}{\text{Y}}$ of X and Y, respectively, then the covariance can be expressed as Eq 11:

$$\text{Cov}(X,Y)=\frac{\sum (X_{i}-\bar{X})(Y_{i}-\bar{Y})}{n-1}$$ (11)

where *X*_*i*_ and *Y*_*i*_ are each value of X and Y, respectively, and n is the amount of data. Calculate the standard deviations $\sigma_{X}$ and $\sigma_{Y}$ of X and Y and the Pearson correlation coefficient (denoted as r), respectively, expressed as Eq 12:

$$r=\frac{\text{Cov}(X,Y)}{\sigma_{X}\times \sigma_{Y}}$$ (12)

The Pearson correlation coefficient is a statistic that measures the degree of linear correlation between two continuous variables. Its value ranges from -1 to 1, where a value close to 1 indicates a strong positive correlation. Values close to -1 indicate a strong negative correlation. Values close to 0 indicate no or weak linear correlation. Thus, the modified Pearson hill climbing algorithm of the camera focus search strategy in the multi-light scene is:

(1) The initial position and the images acquired in different lighting scenes every 5 steps were evaluated, and the order of lighting when the camera was focused was determined according to the Pearson correlation coefficient of the obtained data.

(2) Under the first light source, the search was started with a large step size L, and the starting position, step size L, and 2L images were evaluated, and the sharpness values were F, Fm, and Fe.

(3) Compare the new F, Fm, and Fe, and continue to step 2 if Fs<Fm<Fe. If Fs < Fm and Fe<Fm, step 4 is performed on the range from the focal length of Fs to the interval of Fm (Ls, Lm).

(4) The maximum image sharpness value is obtained by the traversal method for the interval Ls, Lm, and the focal length of the maximum value is F1 at the positive focal point.

(5) Under the second light source, the traversal search is carried out in small steps L’ on the left and right sides of the focal length F1, and the maximum image sharpness value is obtained after comparison, that is, the positive focus position F2.

(6) Perform step 3 to obtain the focal length of the remaining light sources in turn according to the order of the acquired lighting, and complete the camera autofocus in the multi-light source scene.

**Fig 4:**
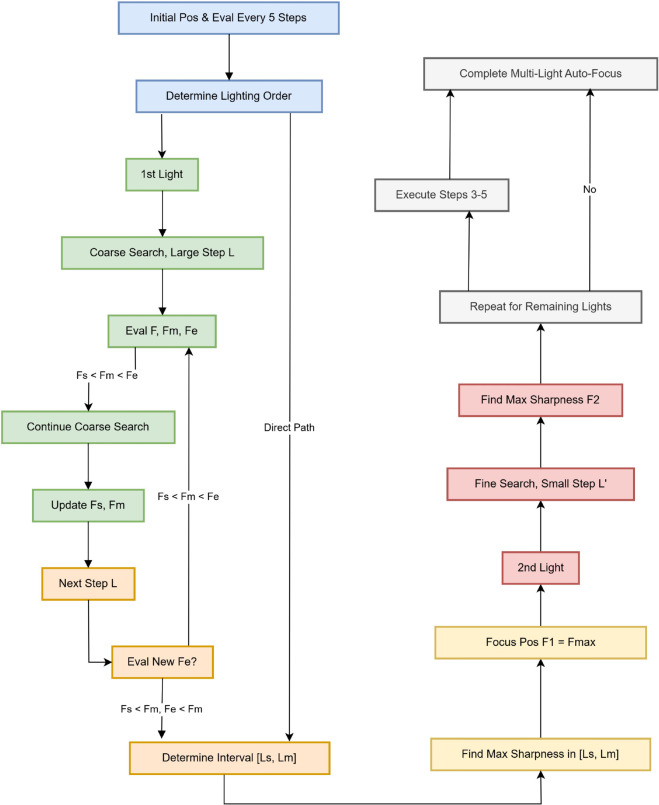
Optimization algorithm combined with the Pearson correlation coefficient.

The optimization algorithm combined with the Pearson correlation coefficient is shown in Fig 4.

## 4 Experimental results and analysis

**Fig 5:**
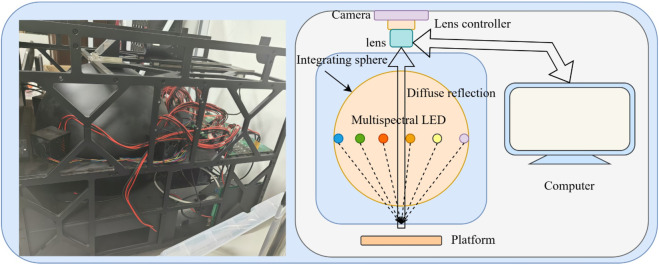
Hardware and schematic diagram of the experimental system.

The experimental system is shown in Fig 5, including an industrial computer, an 8-zone controllable LED light source, a high-definition industrial camera, and a lens controller for zoom. The imaging system employed a Teledyne LT665RM industrial camera with a Sony ICX694 sensor. A Canon EF 50mm f/1.8 STM lens was used for image acquisition. Lens focusing was controlled via a motorized lens controller manufactured by Innovative Scientific Solutions, Inc. (ISSI). The image resolution used in the experiments is 1600 * 1200 pixels. The entire focusing range of the Canon EF50mm f/1.8 STM lens was discretized into a scale of 0–3000. Considering the use of the Pearson correlation coefficient, the focusing strategy for the first spectral band differed from that of the subsequent bands. For the first band, a variable step-size approach was applied across the entire focusing range (0–3000), starting with a coarse step of 100, which was progressively reduced with a decay factor of 0.5, ultimately reaching a step size of 1 to ensure optimal precision. From the second spectral band onward, due to the correlation with the preceding band and the narrower defocusing range, fine focusing with a fixed step size of 1 was directly employed. The analysis of the experimental data was performed on the computer controlling the multispectral device, and SIFT was implemented in the Python OpenCV environment with default parameters.

**Fig 6:**
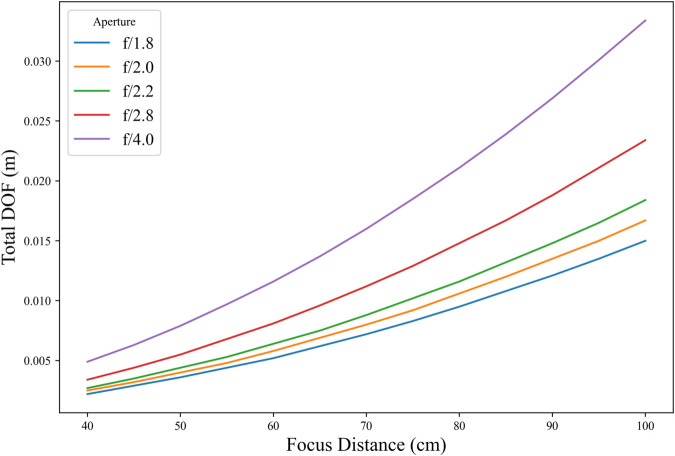
DOF versus focus distance under varying apertures.

To provide a quantitative reference for comparison with the focusing results, the spatial resolution and the optical transfer function (OTF) of the lens under accurate focus were analyzed. The results are presented in Fig 6, where the spatial resolution and modulation transfer function (MTF) curves demonstrate the intrinsic imaging performance of the lens, thereby serving as a baseline for evaluating the proposed focusing method. To verify the advantages of the SIFT Quad-Tenen image sharpness evaluation function in terms of stability and sensitivity, this study compares several common image sharpness evaluation functions through experiments. The selected comparison objects include SMD function, Roberts function, Brenner function and EOG function. In addition, the experiment also includes the traditional Tenengrad function and the improved Quad-Tenengrad function for extracting four-directional gradient information.

**Fig 7:**
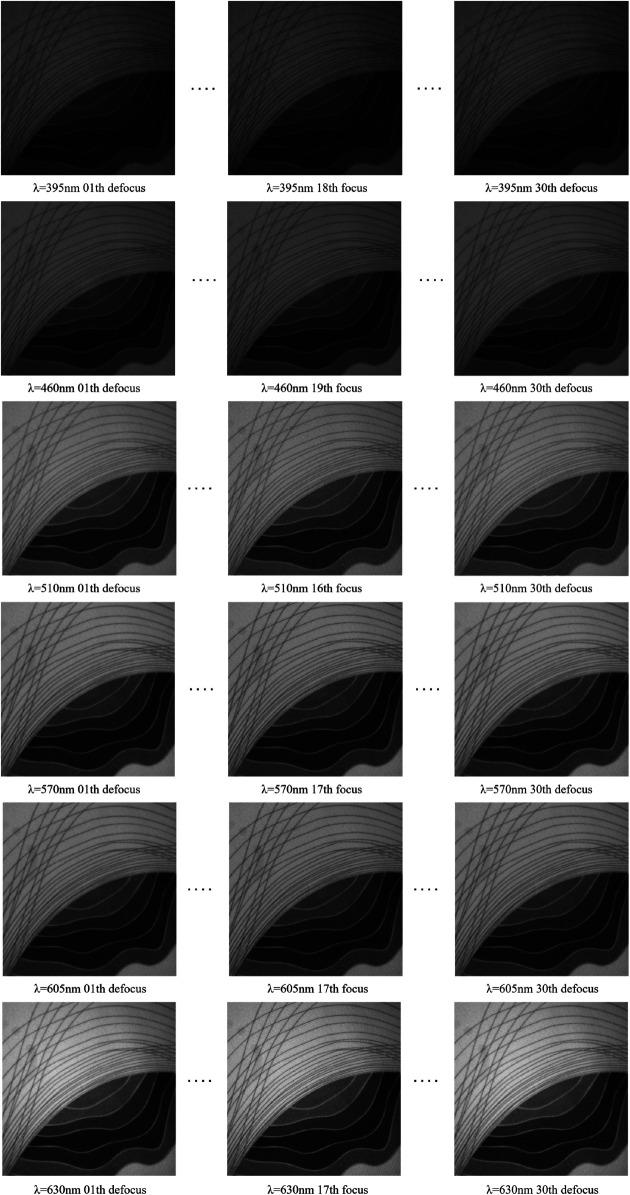
Scene images with multiple light sources.

In the experimental system, the lens was controlled to focus under six different light sources: purple, white, yellow, red, green, and blue, the wavelength ranges of all colors are shown in Table 1. Each set of experiments collected an image sequence from defocus to focus and then back to defocus to comprehensively evaluate the performance of each evaluation function at different focal lengths. The experimental comparisons were all performed on an industrial computer, as shown in Fig 7.

**Table 1:** Wavelength ranges of different colors.

Color	Wavelength (nm)
Purple	380–450
Blue	450–470
Green	495–570
Yellow	570–590
Orange	590–620
Red	620–750

Due to the different dimensions of the image sharpness values evaluated by different definition evaluation functions, to compare the experiments, the maximum value of the image sharpness values of each function is normalized, and the expression is as Eq 13:

$$Z=\frac{F_{i}}{F_{\max}}$$ (13)

Among them, *F*_*i*_ is the sharpness value of the i-th image, and $F_{\max}$ and $F_{\min}$ is the maximum and minimum sharpness value evaluated by the evaluation function for each group of images. The higher the sharpness ratio, the stronger the evaluation function’s ability to distinguish between in-focus images and out-of-focus images.

Experimental comparison requires objective indicators to evaluate different functions. This experiment selected the sharpness ratio R, sensitivity *M*_*SE*_, steepness S, and whether the maximum image sharpness value is correctly evaluated [12]. The formula for the sharpness ratio R is Eq 14:

$$R=\frac{F_{\max}}{F_{\min}}$$ (14)

The sensitivity *M*_*SE*_ formula is Eq 15:

$$M_{se}=\frac{F_{\max}-F_{\max+\Delta x}}{F_{\max}}$$ (15)

$F_{\max+\Delta x}$ represents the sharpness value of the image at point Δx. A greater sensitivity parameter *M*_*SE*_ indicates that the evaluation function is more sensitive in the vicinity of the optimal focus position, allowing for a clearer assessment of the sharpness values near the optimal focus position. The formula for steepness S is as Eq 16:

$$\{\begin{array}{c} S_{R}=\frac{F_{\max}-F_{\max+\Delta x}}{\Delta x}\\ \;S_{L}=\frac{F_{\max}-F_{\max-\Delta x}}{\Delta x}\\ \;S=\frac{S_{R}+S_{L}}{2} \end{array}$$ (16)

*S*_*R*_ denotes the right steepness, and *S*_*L*_ indicates the left steepness. The higher the steepness, the more it indicates that the evaluation function has good sharpness and resolution capability for defocused images near the positive focal position.

By selecting scenes with purple, white, blue, green, yellow, and red light sources to experiment with the improved evaluation function, the following are the evaluation function curves and objective assessments for the six types of light sources, where *α* indicates whether the maximum image sharpness value is correctly assessed.

**Fig 8:**
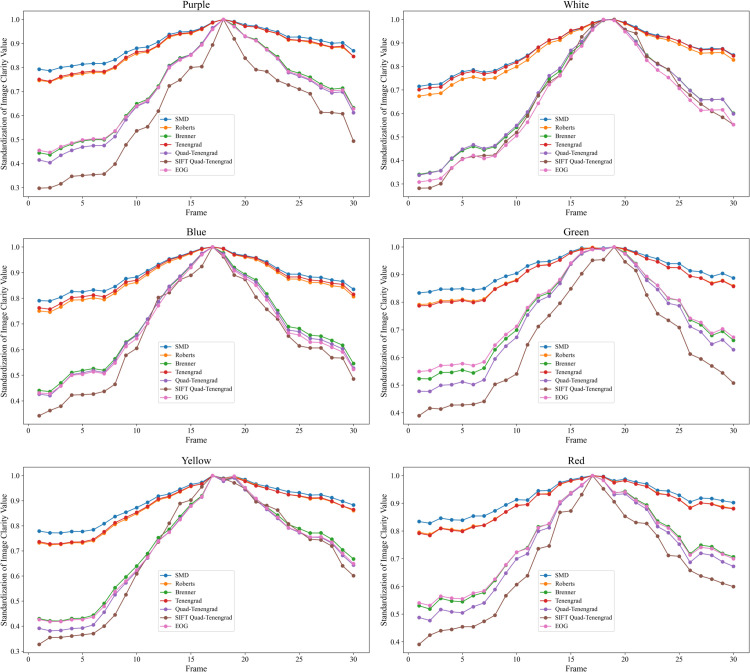
Evaluation parameter performance of each algorithm (In six light scenes).

By analyzing the performance of the image sharpness evaluation function in different light source scenes, it can be observed that the improved SIFT Quad-Tenen evaluation function is compared with the SMD, Roberts, Brenner, EOG function, Tenengrad function, and Quad-Tenengrad function. Significant performance is shown in multiple light scenes as shown in Table 2 and Fig 8. We have selected six lighting scenarios with a wavelength range of 395nm - 630nm, corresponding to lighting colors of purple, white, blue, green, yellow, and red. The specific data show that, at the purple light scenario, the sharpness ratio R shows an improvement of 149.9% compared to the Tenengrad function and 36% over Quad-Tenengrad. The sensitivity *M*_*SE*_ is enhanced by 209.6% relative to the Tenengrad function and 85% compared to Quad-Tenengrad. Additionally, the steepness S demonstrates a 185.7% increase over the Tenengrad function and a 53.8% improvement compared to Quad-Tenengrad. And the SIFT Quad-Tenen exhibits superior performance across all metrics compared to both the Tenengrad function and Quad-Tenengrad.

**Table 2:** Evaluation function metrics under six lighting scenarios.

		SMD	Roberts	Brenner	EOG	Tenengrad	Quad_Tenengrad	SIFTQuad_Tenen
Red	R	1.208	1.269	1.930	1.883	1.275	2.098	2.559
	*M*_*SE*_	0.013	0.018	0.057	0.059	0.018	0.066	0.147
	S	0.006	0.008	0.025	0.026	0.008	0.028	0.047
	*α*	True	True	True	True	True	True	True
Green	R	1.267	1.340	2.290	2.337	1.321	2.378	2.921
	*M*_*SE*_	0.034	0.040	0.107	0.120	0.036	0.113	0.127
	S	0.011	0.014	0.037	0.041	0.012	0.038	0.042
	*α*	True	True	True	True	True	True	True
Blue	R	1.399	1.485	2.936	3.244	1.428	2.964	3.546
	*M*_*SE*_	0.057	0.064	0.152	0.105	0.060	0.155	0.157
	S	0.015	0.018	0.042	0.044	0.016	0.042	0.039
	*α*	True	True	True	False	True	True	True
White	R	1.267	1.340	2.290	2.337	1.321	2.378	2.921
	*M*_*SE*_	0.034	0.040	0.107	0.120	0.036	0.113	0.127
	S	0.011	0.014	0.037	0.041	0.012	0.038	0.042
	*α*	True	True	True	True	True	True	True
Purple	R	1.272	1.352	2.291	2.242	1.348	2.477	3.368
	*M*_*SE*_	0.028	0.032	0.084	0.088	0.052	0.087	0.161
	S	0.018	0.015	0.038	0.040	0.021	0.039	0.060
	*α*	True	True	True	True	True	True	True
Yellow	R	1.296	1.381	2.379	2.391	1.374	2.624	3.054
	*M*_*SE*_	0.016	0.020	0.048	0.045	0.023	0.046	0.057
	S	0.015	0.014	0.035	0.037	0.014	0.038	0.041
	*α*	True	True	True	True	True	True	True

At the white light scenario, the improved evaluation function outperforms the SMD, Roberts, Brenner, and EOG methods in both sharpness ratio and sensitivity. Specifically, its sharpness ratio R is 148.3% higher than the Tenengrad function and 22.5% higher than the Quad-Tenengrad variant. Its sensitivity is 161.7% higher than Tenengrad and 43.4% higher than Quad-Tenengrad. And its steepness S is 143.8% higher than Tenengrad and 40% higher than Quad-Tenengrad. Images acquired at focus and in the near-focus region at a wavelength of white contain more information in both horizontal and vertical directions, but the inclusion of noise leads the EOG function to misidentify the optimal plane. By contrast, the SIFT Quad-Tenen function not only makes correct focus decisions but also preserves outstanding performance in sharpness ratio, sensitivity, and steepness. At the yellow light scenario, the improved evaluation function outperforms the SMD, Roberts, Brenner, and EOG methods in both sharpness ratio and sensitivity. Specifically, compared to the standard Tenengrad function, it increases the sharpness ratio by 122.2%, sensitivity by 147.8%, and steepness by 192.9%; and relative to the Quad-Tenengrad variant, it raises the sharpness ratio by 16.4%, sensitivity by 147.8%, and steepness by 23.9%.

In the red light scenario, the SIFT Quad-Tenen function achieves a 101.1% improvement in sharpness ratio, a 252.8% increase in sensitivity, and a 250% gain in steepness compared to the Tenengrad function. In the green light scene, the corresponding improvements are 121.1%, 252.8%, and 250%, respectively, while in the blue light scene, the SIFT Quad-Tenen function is increased by 148.3% in the sharpness ratio, 161.7% in sensitivity, and 143.8% in steepness. These results demonstrate the superior performance of the SIFT Quad-Tenen function in image sharpness evaluation and reflect its wide applicability under different light source conditions. In addition, through the analysis of the image sharpness curve, it is found that the number of local extrema generated by the SIFT Quad-Tenen function is significantly less than that of other evaluation functions. This means that the SIFT Quad-Tenen function is less susceptible to noise interference during autofocus, reducing the likelihood of false positives. The Brenner and EOG functions mainly extract the information in the horizontal and vertical directions of the image and perform the sum of squares. Still, in the blue light source scene, the images taken by the positive focus and its nearby focal lengths contain a lot of noisy information, which makes the EOG function prone to errors in judging the maximum definition. In contrast, the SIFT Quad-Tenen function can not only maintain a high resolution ratio, sensitivity, and steepness performance while ensuring correct judgment.

The experiments under six light source scenes show that the images collected under different light sources contain different information, and the image sharpness value and focal length change. It can be seen from Table 2 that the SIFT Quad-Tenen evaluation function has good sensitivity under different light sources compared with other evaluation functions, and can accurately find the image with the maximum sharpness value in each scene. The SIFT Quad-Tenen evaluation function has better adaptability and robustness under different light source scenes.

**Fig 9:**
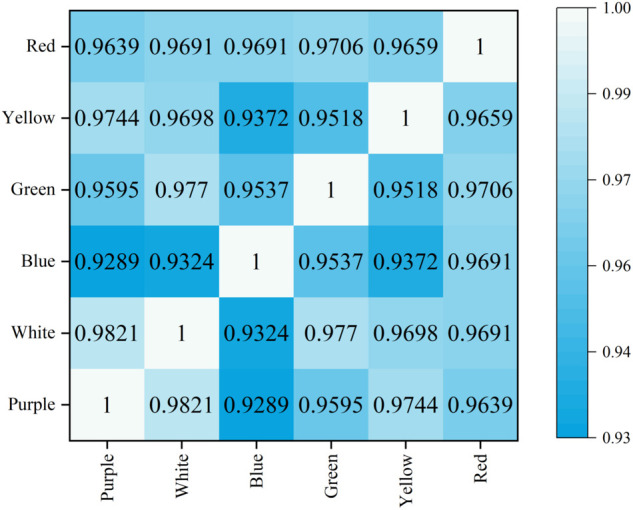
Pearson correlation coefficient.

The autofocus search strategy experiment was carried out in the camera’s multi-light scene. The SIFT Quad-Tenen image sharpness evaluation function was used to evaluate the sharpness of the acquired images. The search strategy was used to search for the optimal focal length position. In the improved hill climbing search algorithm, the Pearson correlation coefficient is obtained by evaluating the initial position and the images collected in different light scenes every 5 steps, and the Pearson correlation coefficient is shown in Fig 9.

By analyzing the Pearson correlation coefficient, it was found that the red light source had the greatest correlation with the white light source. Further observation of the correlation coefficients between red and white light sources and other light sources shows that the correlation between white and yellow light sources is the largest. Based on these findings, this study determined the order in which the camera was focused: red first, white second, and so on. Therefore, the improved search strategy is :

In the initial position and every 5 steps of the search process, the images under different lighting scenes were collected and evaluated, and the lighting sequence of the camera when focusing was determined according to the Pearson correlation coefficient. The order of determination is: red, white, yellow, green, blue, purple. Under the red light source, the improved hill-climbing algorithm is used to search for the positive focus position F-red. Under the blue light source, a traversal search of small steps is carried out with F-red as the center to obtain the maximum image sharpness value, that is, the positive focus position F-white. Focus search for other light sources: Perform step 3 to obtain F-yellow, F-green, F-blue, and F-purple according to the order of the obtained lighting, and complete the camera autofocus in a multi-light source scene.

**Fig 10:**
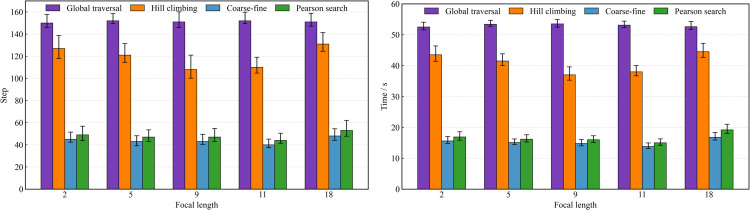
Autofocus search algorithm optimization experiment results.

The experiment was divided into three indicators, namely the focus search step of all light scenes, the focus time, and whether all light source scenes were correctly focused. The experiments were carried out by the traversal method, the hill climbing search algorithm, the coarse-fine hill search algorithm, and the Pearson hill search algorithm after adding the Pearson coefficient, in which the hill climbing search algorithm and the coarse-fine hill search algorithm were experimented with random light source sequences. The initial focal length of the experiment was randomly 0, 5, 8, and 11. As shown in Fig 10, the traditional traversal method leads to too long focusing time due to the comprehensiveness of its search strategy in a multi-light scene.

Although the traditional hill-climbing search algorithm has reduced the focus time, it still does not achieve the expected efficiency. In contrast, the improved coarse-fine hill search algorithm and the Pearson hill search algorithm with the Pearson coefficient significantly reduce the focus time and show better performance. Although the preliminarily improved coarse hill search algorithm is improved considerably compared with the traditional hill climbing search algorithm in terms of focus time, it still has limitations in dealing with large focal length gaps in different light source scenes. When the focal lengths of two light sources differ significantly and they are focused sequentially in the order in which they are lit, one of the light sources may fail to focus. To solve this problem, the Pearson climbing search algorithm is introduced, which uses the Pearson correlation coefficient to guide the search process by considering the correlation between different light sources. This method not only optimizes the focusing speed but also improves the accuracy of focusing, ensuring that the camera can quickly and accurately complete focusing in multi-light source scenes. The experimental results have verified the superiority of the Pearson hill climbing search algorithm in terms of focusing speed and accuracy in multi-light source scenes. By combining the image sharpness evaluation function with an improved search strategy, this study provides an effective solution for the application of autofocus technology under different light sources.

## 5 Conclusion

This study proposes a new image sharpness evaluation function, SIFT Quad-Tenen function, to address the problem of camera autofocus in multi-light source scenes in multispectral imaging systems, to improve the ability to accurately evaluate captured images under multi-light source conditions. This function introduces the extraction of multi-directional image information based on the traditional Tenengrad function, enhancing the accuracy of evaluation. At the same time, considering the characteristics of the SIFT algorithm in terms of illumination invariance, it integrates it into the evaluation function, which effectively overcomes the influence of different light sources. To further improve the focusing speed and accuracy, the hill climbing algorithm was improved, and the Pearson hill climbing algorithm was proposed. While maintaining the advantages of fast coarse focusing of the hill climbing algorithm, the algorithm introduces the Pearson correlation coefficient to realize the fine adjustment of the focusing process according to the characteristics of multi-light source scenes, to ensure the accuracy of camera focusing. Through experiments, we verified the cooperation between the SIFT Quad-Tenen image sharpness evaluation function and the Pearson Hill climbing algorithm, and successfully solved the problem of accurate measurement and fast focusing in the multispectral imaging system. This result not only improves the efficiency of the focus but also ensures the quality of focus in complex lighting environments. In summary, this study provides an effective solution to the problem of autofocus of a multispectral imaging system under different light sources. Through the improved image sharpness evaluation function and the optimized search algorithm, the method in this paper improves the focusing speed and ensures the accuracy of focusing while improving the autofocus under different light sources.
